# A B7-H3–Targeted CD28 Bispecific Antibody Enhances the Activity of Anti–PD-1 and CD3 T-cell Engager Immunotherapies

**DOI:** 10.1158/1535-7163.MCT-24-0327

**Published:** 2024-09-20

**Authors:** Gregory L. Moore, Veronica G. Zeng, Juan E. Diaz, Christine Bonzon, Kendra N. Avery, Rumana Rashid, Jing Qi, Dong Hyun Nam, Jonathan Jacinto, Matthew A. Dragovich, Yoon Kyung Kim, Karen P. Balcazar, Charles G. Bakhit, Araz Eivazi, Hanh Nguyen, Umesh S. Muchhal, David E. Szymkowski, John R. Desjarlais, Michael Hedvat

**Affiliations:** Xencor, Inc., Pasadena, California.

## Abstract

T-cell activation is a multistep process requiring T-cell receptor engagement by peptide–MHC complexes (Signal 1) coupled with CD28-mediated costimulation (Signal 2). Tumors typically lack expression of CD28 ligands, so tumor-specific Signal 1 (e.g., neoepitope presentation) without costimulation may be ineffective or even induce T-cell anergy. We designed the bispecific antibody XmAb808 to co-engage the tumor-associated antigen B7-H3 with CD28 to promote T-cell costimulation within the tumor microenvironment. XmAb808 costimulation was measured by its ability to activate and expand T cells and enhance T cell–mediated cancer cell killing in cocultures of human peripheral blood mononuclear cells and cancer cells and in mice engrafted with human peripheral blood mononuclear cells and tumor xenografts. XmAb808 avidly bound cancer cells and stimulated IL2 and IFNγ secretion from T cells cocultured with cancer cells engineered to deliver Signal 1 to T cells via a surface-expressed anti-CD3 antibody. XmAb808 enhanced expression of the antiapoptotic factor Bcl-xL and CD25, promoting survival and IL2-dependent expansion of T cells coupled with increased T cell–mediated cytotoxicity *in vitro*. XmAb808 combined with an EpCAM×CD3 bispecific antibody to enhance target cell killing through IL2-dependent expansion of CD25^+^ T cells. This combination also suppressed pancreatic tumor xenograft growth in mice. Furthermore, XmAb808 combined with an anti–programmed cell death protein 1 antibody to suppress breast tumor xenograft growth in mice. XmAb808 as monotherapy and in combination with an anti–programmed cell death protein 1 antibody is currently in clinical development in patients with advanced solid tumors. Our results suggest that XmAb808 may also combine with tumor antigen–targeted anti-CD3 (Signal 1) T-cell engagers.

## Graphical Abstract

**Figure:**
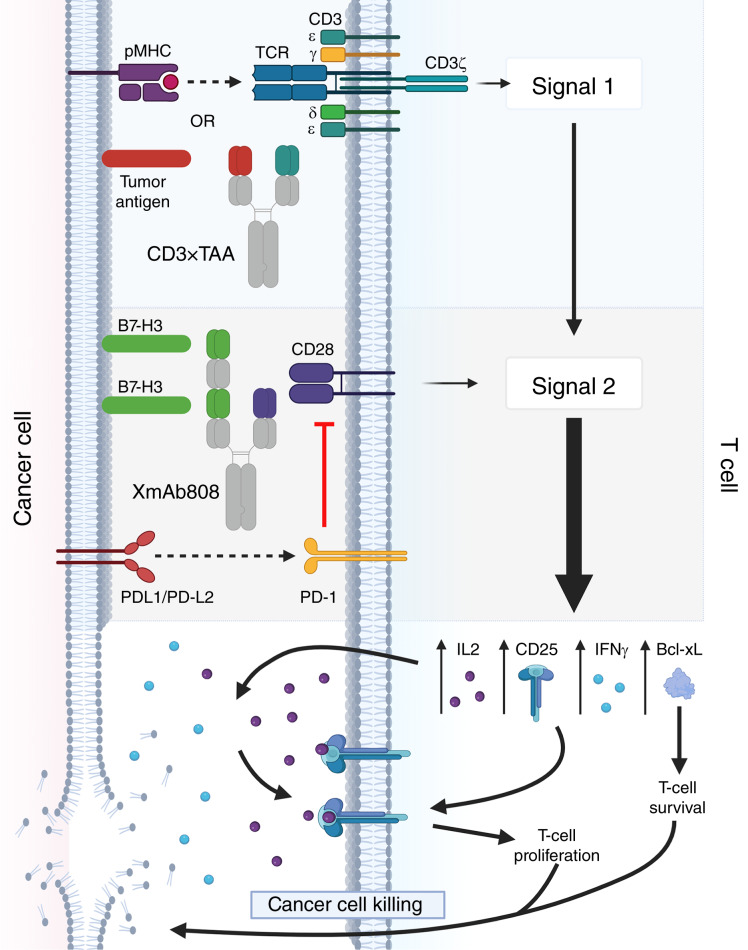

## Introduction

In the first stage of T-cell activation (Signal 1), professional antigen-presenting cells (APCs) present cognate peptide–MHC complexes (pMHCs) to engage T-cell receptors (TCRs). However, Signal 1 alone is insufficient to stimulate strong immune responses and instead can lead to T-cell anergy (1). For optimal immune synapse formation between T cells and APCs, and subsequent robust immune activation, a costimulatory signal (Signal 2) is required. The prototypical costimulatory pathway involves induction of CD28 signaling in T cells by ligands CD80 (B7.1) and CD86 (B7.2), which are predominantly expressed on professional APCs (2). Importantly, cancer cells can deliver Signal 1 to tumor-infiltrating lymphocytes (TILs) through MHC presentation of neoantigenic peptides yet are unable to deliver costimulatory Signal 2 because, like most epithelial cells, they typically lack expression of CD80 or CD86. Although such Signal 1–primed TILs are destined to become anergic (1), this fate might be overcome by introducing targeted costimulation in the tumor microenvironment (TME). Indeed, the crucial role of costimulation in antitumor immunity was first demonstrated over 30 years ago, when artificial expression of CD28 ligands on melanoma cells was shown to eradicate solid melanoma tumors implanted in mice and also created a sustained memory response protecting mice even when re-engrafted with parental tumors lacking costimulatory ligands (3, 4).

The first therapeutic molecule intended to enhance costimulation was TGN1412 (theralizumab), a bivalent anti-CD28 antibody with superagonistic properties; i.e., it activates T cells independently of TCR engagement. TGN1412 promoted strong systemic cytokine release in healthy volunteers (5), a clinical scenario which stalled further development of CD28-targeted therapeutics. More recently, a significant advance in harnessing costimulation as a cancer immunotherapy has been the development of nonsuperagonistic and tumor-targeted CD28 bispecific antibodies that require a separate tumor-associated Signal 1 for efficacy (6–9). These bispecific antibodies monovalently engage tumor antigens to promote clustering of CD28 at the immune synapse, leading to potent T-cell costimulation (7, 10). However, the mechanisms by which such bispecific antibodies enhance antitumor immunity are not fully characterized. Now, we further explore the underlying mechanisms of tumor-targeted costimulation using a novel bispecific antibody that co-engages CD28 with the tumor-associated antigen B7-H3, assessing its activity in combination with endogenous and exogenous sources of Signal 1, and with immune checkpoint inhibition.

B7-H3 (CD276) is a cell surface protein that is overexpressed in a wide range of solid tumors. Numerous therapies targeting B7-H3^+^ cancers have advanced to clinical trials, including antibody–drug conjugates, CAR-T cells, and CD3 T cell–engaging bispecific antibodies. To date, no B7-H3–targeted therapies have been approved, and several trials have been terminated, some with toxicities possibly caused by low-level B7-H3 expression in normal tissues (11). Because Signal 2 activation without Signal 1 is thought to be immunologically inert, we reasoned that a B7-H3–targeted costimulatory antibody would enhance TIL-mediated immunity yet avoid deleterious on-target off-tumor (OTOT) effects likely problematic for other B7-H3–targeted therapies. Our antibody engineering strategy generated the clinical candidate XmAb808, a B7-H3×CD28 bispecific antibody incorporating a nonsuperagonistic, monovalent CD28-binding domain coupled with a high-avidity, bivalent B7-H3–binding domain.

Here, we demonstrate that XmAb808 induces CD28-mediated T-cell costimulation in a tumor antigen–dependent fashion. XmAb808 promoted IFNγ release from T cells and IL2-mediated expansion of T cells with enhanced survival, translating to increased T cell–mediated killing of cancer cells *in vitro* but only in the presence of both Signal 1 and B7-H3^+^ target cells. Furthermore, XmAb808 synergized with a targeted Signal 1 stimulus (a CD3-engaging bispecific) to completely suppress tumor xenograft growth. Finally, because programmed cell death protein 1 (PD-1) is known to inhibit CD28 signaling (12, 13), we combined XmAb808 with an anti–PD-1 antibody to increase its antitumor efficacy against human tumor xenografts in mice.

## Materials and Methods

### Antibodies

Phage display libraries were designed using human antibody repertoire sequence data and panned against the extracellular domains of B7-H3 and CD28 to identify B7-H3–specific and CD28-specific antibodies. The libraries contained minimal light-chain diversity, enabling the use of a common light chain compatible with both B7-H3 and CD28 heavy chains. Both B7-H3 and CD28 antibodies were then affinity-matured by phage display using secondary libraries comprising only heavy chain diversity.

Bispecific antibodies were generated as previously described (14). XmAb808 [sequence ID numbers 2019, 2020, and 2021, published in US11591401 (15)] was configured in a Fab_2_–Fab–Fc common light-chain format with duplicate B7-H3 Fab domains in a stacked arrangement connected by a 10–amino acid linker. The human IgG1 constant region contained substitutions to minimize antibody-mediated effector functions, promote heterodimerization, facilitate purification, and enhance *in vivo* half-life. To produce XmAb808, three plasmids containing heavy chain 1: CD28 Fab–Fc, heavy chain 2: B7-H3 Fab_2_–Fc, and the common light chain: B7-H3/CD28 were co-transfected into HEK293E cells (National Research Council) with 293fectin (12347500; Thermo Fisher Scientific). Media were harvested 5 days after transfection, and protein was purified from the supernatants with MabSelect Protein A resin (17519903; Cytiva Life Sciences). The protein pool from the protein A step was then further purified with an NGC Quest 10 plus cation-exchange chromatography system (Bio-Rad Laboratories) to isolate XmAb808 from any product-related impurities. Protein was injected onto a HiTrap SP HP 16 × 25 mm (5.0 mL) column (Cytiva Life Sciences) at 0.5 mL/minute and eluted at 3 mL/minute with 50 mmol/L 2-(N-Morpholino)ethanesulfonic acid pH 6.0 as the mobile phase and 50 mmol/L MES pH 6.0 plus 1 mol/L NaCl as the elution phase. A linear gradient from 5% to 40% of the elution phase over 60 minutes was used. After CIEX purification, XmAb808 was buffer-exchanged into PBS, concentrated, and characterized by analytical-scale size-exclusion chromatography and analytical-scale CIEX using an Agilent 1200 high-performance liquid chromatography system (Agilent Technologies).

XmAb808^s^ is an analogue of XmAb808 that does not contain the *in vivo* half-life–enhancing substitutions. Mono–B7-H3×CD28 was constructed similarly to XmAb808 but omitted the N-terminal B7-H3 Fab domain, resulting in its monovalent binding to B7-H3. B7-H3×Null was constructed similarly to XmAb808^s^, but the CD28 variable heavy-chain region was replaced with that of the anti–respiratory syncytial virus antibody motavizumab (16); the resulting variable region lacked binding to both CD28 and respiratory syncytial virus. EpCAM (3622W94)×CD3 (17) and B7-H3×CD3 were configured in a Fab–single-chain variable fragment (scFv)–Fc format incorporating an anti-CD3ϵ–binding domain with a CD3 affinity of 7 to 15 nmol/L. The B7-H3–binding domain of this B7-H3×CD3 bispecific antibody does not compete with XmAb808 for binding. The anti–PD-1 antibody was generated by subcloning the Fv domain of nivolumab into a human constant region containing substitutions to minimize antibody-mediated effector function. TGN1412 was derived from the published patent (18) and produced at Xencor.

### Biolayer interferometry

Binding of XmAb808 to human (Sino Biological; 90182-C08H) and mouse (R&D Systems; 483-CD-200/CF) CD28 was measured using an Octet HTX instrument (Sartorius). CD28 proteins were captured on anti–His tag (HIS1K; Sartorius) biosensors for 3 minutes at 20 or 40 nmol/L. Buffer-loaded sensors and a buffer-only analyte sample were included for double reference subtraction. Biosensors were then dipped into a three fold serial-dilution series of XmAb808 starting from 3,000 nmol/L for a 5-minute association phase, followed by a 5-minute dissociation phase in buffer. Each dataset was collected in triplicate at 25°C. HBS-EP+ with 0.5% BSA was used for sample buffer, and 10 mmol/L glycine pH 2.0 was used for biosensor regeneration. Data were processed and fit using Data Analysis HT software version 11.1.3.50. Data were double reference–subtracted, y-aligned to the first 5 seconds of the baseline, interstep-corrected to dissociation, and Savitsky–Golay–filtered. Data series were globally fit to a 1:1 Langmuir model including the full association phase and the first 30 seconds of the dissociation phase.

### In-tandem epitope-binning assay

In-tandem-style bidirectional epitope-binning studies were performed using an Octet HTX instrument (Sartorius). Human CD28-Fc-His-AviTag protein (produced at Xencor) was captured at 20 nmol/L for 2 minutes onto anti-His tag (HIS1K, Sartorius) biosensors. Each biosensor was then dipped first into 500 nmol/L XmAb808 followed by 500 nmol/L TGN1412 or first into 500 nmol/L TGN1412 followed by 500 nmol/L XmAb808 so that the antibody pair was observed in both directions. Data were analyzed using the manufacturer’s software.

### Cell lines

HEK293, MDA-MB-231, HPAF-II, A431, and 22Rv1 were purchased from ATCC (CRL-1573, RRID: CVCL_0045; HTB-26, RRID: CVCL_0062; CRL-1997, RRID: CVCL_0313; CRL-1555, RRID: CVCL_0037; and CRL-2505, RRID: CVCL_1045; ATCC); LOX-IMVI from Sigma-Aldrich (SCC201; RRID: CVCL_1381); and A431-β2-microglobulin (β2M)-null cells from Abcam (ab261893; RRID: CVCL_B1BS). HEK293, MDA-MB-231, HPAF-II, A431, A431-β2M, and 22Rv1 cell lines were purchased in 2016, 2015, 2017, 2021, 2021, and 2019, respectively. Cell lines were confirmed to be *Mycoplasma* negative by PCR, and their identities were authenticated by genotyping (IDEXX BioAnalytics): MDA-MB-231 cells were tested on January 21, 2022; HPAF-II on July 14, 2023; A431 on June 23, 2022; 22Rv1 on January 3, 2024; and LOX-IMVI on January 3, 2024. HEK293 cells were not tested for *Mycoplasma* contamination, and cell line identity was not authenticated. Cell lines exceeding 20 passages were not used. To generate cell lines that stably express membrane-bound anti–CD3ϵ scFvs, we transfected cells with anti–CD3ϵ (SP34) scFv DNA using Lipofectamine 2000 (Invitrogen). The anti–CD3ϵ scFv domain has been characterized to have a K_D_ of 3.7 nmol/L and was anchored to the cell membrane using a single-pass transmembrane domain. Positive clones were identified using an anti-idiotypic CD3 primary antibody (Xencor) and an anti–rabbit IgG-Fc fragment secondary antibody (Jackson ImmunoResearch Laboratories, Inc.; 111-605-046; RRID: AB_2338076).

B7-H3 expression in HEK293-αCD3 cells was suppressed [HEK293-αCD3-B7-H3 knockout (KO)] using a TALEN/CRISPR-Cas9 expression plasmid (GeneCopoeia; HCP219700-CG04-3-10-c) containing single guide RNAs targeting B7-H3 (GCT​GCA​GCG​CGT​GCG​TGT​GG). B7-H3 expression was determined by flow cytometry (BD FACSymphony A3 Cell Analyzer; BD Biosciences) with a PE-conjugated anti–human B7-H3 antibody (BioLegend; 331606; RRID: AB_1279197; DCN.70) and Quantum Simply Cellular anti–human IgG beads (Bangs Laboratories, Inc.; 816-B), according to the manufacturer’s protocol.

A431-NLV and A431-G209 cells were generated by stably expressing a fusion of β2M and human leukocyte antigen (HLA)-A*0201 fused to the cytomegalovirus (CMV) pp65–derived peptide antigen NLVPMVATV (NLV) or the human melanoma gp100–derived peptide antigen IMDQVPFSV (G209), respectively, in A431-β2M-null cells. β2M–HLA-A*0201 fusion vectors were PCR-amplified and assembled as previously reported (19). HLA-A2 expression was measured as a surrogate for expression of the fusion proteins using a PE-labeled HLA-A2 antibody (BioLegend; 343306; RRID: AB_1877227; BB7.2), and B7-H3 expression was verified with a PE-labeled anti–human B7-H3 antibody; both were assessed using flow cytometry. A431-NLV and A431-G209 clones with equivalent HLA-A2 and B7-H3 expression were selected.

To generate MDA-MB-231-pp65 cell lines, we transfected MDA-MB-231 cells with a lentivirus delivering CMV structural protein pp65 (GeneCopoeia; LPP-CS-GS195T-Lv183-01-400). Stable clones were selected with neomycin, and pp65 expression was confirmed by intracellular staining with an anti-pp65 antibody (Thermo Fisher Scientific; MA1-7597; RRID: AB_1073982).

22Rv1–Nuclight-red (NLR) cells with fluorescently labeled nuclei were generated for real-time cell killing assays by infection with Incucyte Nuclight Red Lentivirus (Sartorius; 4476) and grown stably under selection.

### Antibody binding

Cells were incubated with engineered antibodies and then stained with Alexa Fluor 647 Goat Anti-Human IgG (Jackson ImmunoResearch Laboratories, Inc.; 109-605-170; RRID: AB_2810901), each at 4°C for 1 hour. Data were acquired using a FACSymphony A3 Cell Analyzer.

### Cytokine release assay

XmAb808, TGN1412, and OKT3 (BioLegend; 317325; RRID: AB_11147370) were air-dried to assess superagonism as previously described (20). Briefly, 10 μg of antibodies was dried in a SpeedVac for 2 hours and then incubated with 200,000 human peripheral blood mononuclear cells (PBMCs) for 24 hours; cytokines were measured with an electrochemiluminescence (ECL) immunoassay [Meso Scale Discovery (MSD); K15049D]. Statistical significance was determined using Kruskal-Wallis tests followed by Dunn's *post hoc* tests in GraphPad Prism (RRID: SCR_002798).

### CMV recall assay

Target cells were cocultured with negatively enriched T cells (STEMCELL Technologies; 19051) from a CMV-seropositive donor at an effector to target cell (E:T) ratio of either 25:1 or 10:1 and treated with an XmAb808 dose titration. The supernatants were collected after 24 hours for cytokine measurements using ECL immunoassays (MSD). CMV-reactive CD8^+^ T cells were measured by staining PBMCs with HLA-A*0201/NLV peptide tetramers (MBL International; TB-0010-1).

### T-cell activation and T cell–mediated cytotoxicity

Target cells cocultured with human CD3–enriched T cells were treated with combinations of XmAb808, EpCAM×CD3, B7-H3×CD3, IL2-neutralizing, IFNγ-neutralizing (R&D Systems; 25718 and 5334), and isotype control antibodies (indicated in figure legends). After 1 day, the supernatants were collected to measure cytokines using an ECL immunoassay (MSD). After 1 day for 22Rv1-αCD3 and after 1 and 5 days for 22Rv1-NLR cocultures, T-cell counts and activation were assessed via flow cytometry. T cells were stained with Zombie Aqua viability dye, treated with fluorophore-conjugated anti-CD3, anti-CD4, anti-CD8a, and anti-CD25 (BioLegend; UCHT1, OKT4, HIT8a, and BC96) antibodies, then treated with fixation/permeabilization buffer (eBioscience; 00-5523-00), and stained with fluorophore-conjugated anti–Bcl-xL (Cell Signaling Technology; 2767S; RRID: AB_2274763; 54H6). Data were analyzed using FlowJo version 10.7.1 (RRID: SCR_008520). 22Rv1-αCD3 viability was monitored using an xCELLigence Real-Time Cell Analysis System (Agilent Technologies), and 22Rv1-NLR viability was monitored using the Incucyte Live-Cell Analysis System (Sartorius).

To measure IL2^+^ T cells by flow cytometry, we stained T cells with anti-CD3, anti-CD4, anti-CD8, anti-CD45RA, and anti-CCR7 (BioLegend; UCHT1, OKT4, RPA-T8, HI100, and G043H7, respectively) antibodies, then treated cells with fixation/permeabilization buffer, and stained with a fluorophore-conjugated anti-IL2 antibody (BioLegend; MQ1-17H12).

Chronically stimulated T cells were generated by incubating CD3-enriched T cells with anti-CD3 and anti-CD28 Dynabeads (Thermo Fisher Scientific) for 1 week with 10 ng/mL IL2 (PeproTech). Culture medium was changed every 2 days, adding fresh Dynabeads and IL2. To characterize phenotypes of resting and stimulated T cells, we stained T cells with Zombie Aqua viability dye and fluorophore-conjugated anti-CD3, anti-CD4, anti-CD8, anti–PD-1, anti-TIM3, anti-LAG3, and anti-CTLA4 (BioLegend; UCHT1, OKT4, RPA-T8, EH12.2H7, F38-2E2, 11C3C65, and l3D10) antibodies. We also stained T cells with fluorophore-conjugated anti-TOX antibody (BD Biosciences; 568356; NAN448B) following fixation and permeabilization to detect intracellular staining.

### Mouse xenograft studies

Xencor’s Institutional Animal Care and Use Committee approved all mouse procedures, which adhered to the United States Department of Agriculture (USDA) Animal Welfare Act and the Guide for the Care and Use of Laboratory Animals (21). Immunocompromised female NSG-MHC I/II DKO mice (The Jackson Laboratory; strain 025216; RRID: IMSR_JAX:025216) were inoculated intradermally with 1 × 10^6^ HPAF-II or 3 × 10^6^ MDA-MB-231-pp65 cells at 7 to 9 weeks of age. Following the formation of palpable tumors, mice were assigned to treatment groups, engrafted with 5 × 10^6^ human PBMCs intraperitoneally, and injected with weekly treatment intraperitoneally. Tumors were measured using calipers every 2 to 4 days; approximate tumor volumes (mm^3^) were calculated using the formula 0.5 (length × width^2^).

Blood samples were collected via retro-orbital sinus puncture of isoflurane-anesthetized mice to measure peripheral leukocytes. Whole blood was treated with Fc receptor blockers (BioLegend; 101339; RRID: AB_2616683 and 422302; RRID: AB_2818986) and then stained with fluorophore-conjugated anti-hCD45 (BD Biosciences; 2D1), anti-hCD4, and anti-hCD8a (BioLegend; RPA-T4 and RPA-T8) antibodies for 1 hour at 4°C, followed by erythrocyte lysis with ACK lysis buffer. Cells were fixed in 1% paraformaldehyde and analyzed with flow cytometry.

Mice were monitored daily and euthanized if needed according to ethical standards. Several mice were euthanized because of excessive HPAF-II tumor volumes: six PBS-treated mice after day 28 and one EpCAM×CD3–treated and two EpCAM×CD3 plus B7-H3×Null–treated mice after day 29. In the MDA-MB-231-pp65 study, two mice were euthanized: one PBS-treated mouse on day 47 because of large tumor volume and one XmAb808-treated mouse on day 46 because of hind limb paralysis. For these mice, the final tumor volume measurements were carried forward through subsequent timepoints.

Sample sizes were the minimum needed to determine antitumor effects of XmAb808 based on previous experience and established conventions. Statistical differences in tumor volumes and leukocyte counts between groups were determined using Kruskal-Wallis tests followed by Dunn's *post hoc* tests in GraphPad Prism.

### Data availability

All data are available upon reasonable request.

## Results

### XmAb808 binds avidly to B7-H3^+^ cancer cells

**Figure 1.**
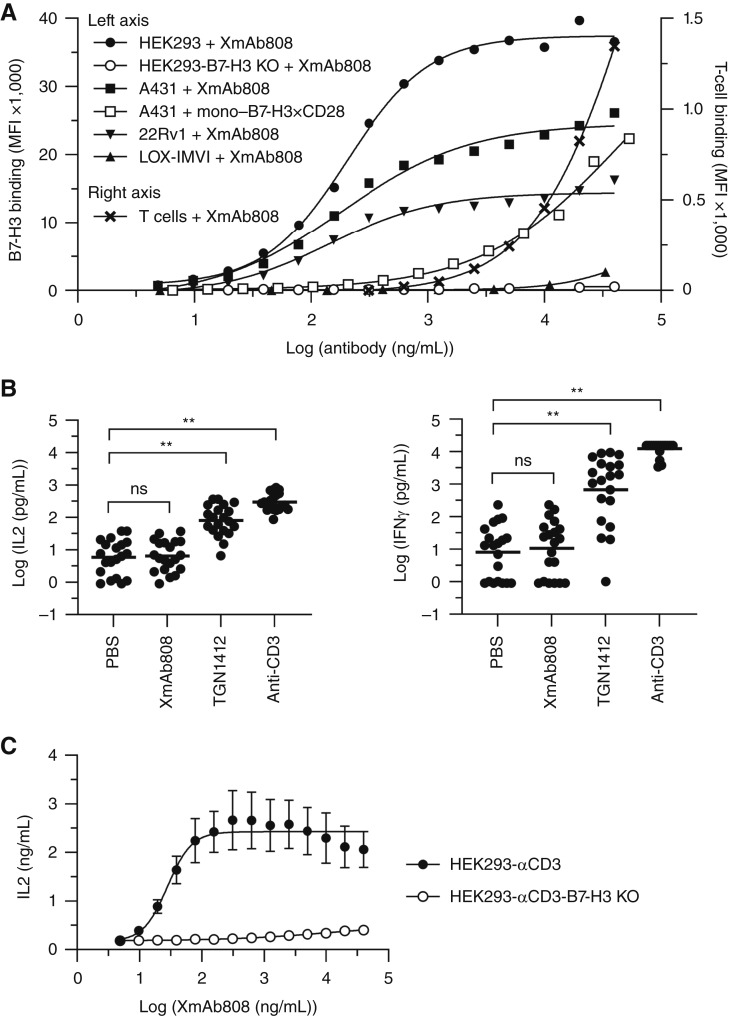
XmAb808 binds avidly to B7-H3 on target cells to costimulate T cells. **A,** Binding of XmAb808 or its analogue containing a single B7-H3–binding domain (mono–B7-H3×CD28) to target cells with varying B7-H3 densities (left axis) and binding of XmAb808 to human T cells (right axis) are plotted as mean fluorescence intensity (MFI). HEK293 cells express approximately 200,000 B7-H3 antigens; A431, 196,000; 22Rv1, 86,000; LOX-IMVI cells do not express B7-H3. Because mono–B7-H3×CD28 has a lower molecular weight than XmAb808 (145 vs. 192 kDa), the mono–B7-H3×CD28 concentrations in this figure were corrected by a factor of 1.3-fold to adjust for molarity. **B,** Human PBMCs from 20 different donors were incubated on plates coated with air-dried (immobilized) XmAb808, anti-CD28 (TGN1412), or anti-CD3 (OKT3) antibodies. IFNγ and IL2 were measured after 24 hours. Statistical significance is denoted by **, *P* < 0.01; ns, not significant. **C,** IL2 release from PBMCs cocultured with HEK293-αCD3 or HEK293-αCD3-B7-H3 KO cells at an E:T ratio of 25:1 was measured after 24 hours. Data are represented as means ± SEM of cocultures with PBMCs from eight different human donors.

To investigate the binding characteristics of XmAb808, a bispecific antibody comprising two B7-H3–binding domains and a single CD28-binding domain, we first focused on its interaction with B7-H3. To determine B7-H3 binding specificity, we compared HEK293 cells that express 200,000 B7-H3 antigens with cells with CRISPR-mediated deletion of B7-H3 (HEK293-B7-H3 KO). XmAb808 bound dose-dependently to HEK293 but not to HEK293-B7-H3 KO cells (Fig. 1A). We also profiled XmAb808 binding to cancer cell lines for use in immune response assays, including A431 epidermoid cancer cells, 22Rv1 prostate cancer cells, and LOX-IMVI melanoma cells, expressing 196,000, 86,000, and no B7-H3 antigens, respectively. XmAb808 bound nearly equipotently to A431, 22Rv1, and HEK293 cells (EC_50_: 184, 144, and 202 ng/mL, respectively); maximal binding depended on B7-H3 antigen density, with no binding to LOX-IMVI cells (Fig. 1A).

To assess the contribution of antigen avidity to XmAb808 binding, we engineered an XmAb808 analogue with a single B7-H3–binding domain (mono–B7-H3×CD28). This antibody bound A431 cells >100-fold less potently (Fig. 1A), suggesting that the bivalent format of XmAb808 drives avid binding to cancer cells expressing higher B7-H3 levels relative to normal cells with lower levels.

In contrast to its high-avidity, bivalent B7-H3 binding, XmAb808 was designed to bind T cells via a monovalent, lower-affinity CD28-binding domain to preclude CD28 clustering on T cells in the absence of B7-H3 and thus avoid undesirable nontargeted T-cell costimulation. To assess this possibility, we examined XmAb808 binding to T cells enriched from human PBMCs. XmAb808 bound T cells with >100-fold lower potency than B7-H3^+^ HEK293, A431, and 22Rv1 cells (Fig. 1A). Taken together, these T-cell and cancer-cell binding results suggest that XmAb808 sequentially engages its two targets, first by avid and high-affinity binding to B7-H3, followed by binding to CD28, to form an artificial immunologic synapse between T cells and B7-H3^+^ cancer cells.

### XmAb808 is not a superagonist

Given the troubling precedent that the bivalent, high-affinity anti-CD28 antibody TGN1412 stimulates uncontrolled T-cell activation in humans (5, 20), we designed XmAb808 to eliminate any potential T-cell costimulation in the absence of targeted TCR engagement. We accomplished this by generating a nonsuperagonistic CD28-binding antibody that differs from the Signal 2 superagonist TGN1412, by making the CD28-binding domain monovalent and relatively low affinity and by silencing the Fc region to eliminate potential CD28 cross-linking by Fcγ receptor–expressing cells. We measured the binding affinity of XmAb808 for CD28 using biolayer interferometry and found that it bound to human CD28 with a K_D_ of 213 nmol/L. XmAb808 also cross-reacts with mouse CD28, a property not observed with TGN1412 (22), suggesting distinct binding epitopes for each antibody. Epitope-binning experiments showed that XmAb808 partially competes with TGN1412 for binding to CD28, further indicating that XmAb808 binds to a nonidentical epitope on CD28 as compared with TGN1412.

To assess its potential for nonspecific T-cell activation, we measured XmAb808 activity in an *in vitro* assay in which TGN1412 is known to stimulate a strong inflammatory cytokine response via hyper–cross-linking of CD28 on T cells (20). XmAb808 and positive controls TGN1412 and OKT3 (a CD3–cross-linking antibody that activates Signal 1) were immobilized by air-drying and subsequently incubated with human PBMCs. In agreement with previous reports (20, 23), TGN1412 stimulated IL2 and IFNγ production from human PBMCs, by 36-fold and 1,048-fold relative to PBS, respectively (Fig. 1B). Likewise, OKT3 also stimulated IL2 and IFNγ production, by 85-fold and 4,516-fold, respectively. In marked contrast, XmAb808 immobilized by air-drying did not promote IL2 or IFNγ secretion from human PBMCs. This cytokine release assay indicates that XmAb808 does not activate human T cells in the absence of Signal 1; therefore, to assess its Signal 2–stimulating properties, we engineered multiple cancer cell lines to provide homogeneous and well-defined Signal 1 to T cells.

### XmAb808 requires surface B7-H3 expression on cancer cells to costimulate T cells

To evaluate XmAb808 costimulation and its dependence on B7-H3 expression, we cocultured HEK293 or HEK293-B7-H3 KO cells with human PBMCs and measured IL2 secretion as a marker of CD28 agonism in T cells (24). We introduced an anti–CD3 scFv on the surface of these HEK293 cells (HEK293-αCD3) as a source of high-affinity Signal 1 (K_D_: ∼4 nmol/L for CD3). Every target cell in this experimental system expresses the same anti-CD3ε–binding domain, ensuring consistent, high-affinity, pMHC-independent engagement of T cells through their TCRs. XmAb808 triggered robust IL2 release from these cocultures in a dose-dependent manner (Fig. 1C). However, despite the strong Signal 1 delivered by HEK293-αCD3 cells, cocultured PBMCs did not produce IL2 in the absence of XmAb808. Although XmAb808 bound T cells at concentrations above 1 μg/mL even in the absence of B7-H3^+^ target cells, presumably through weak monovalent CD28 engagement (Fig. 1A), this binding did not stimulate IL2 release in HEK293-αCD3-B7-H3 KO cocultures (Fig. 1C). This indicates that B7-H3 surface expression is required for XmAb808 to trigger costimulation of T cells.

**Figure 2.**
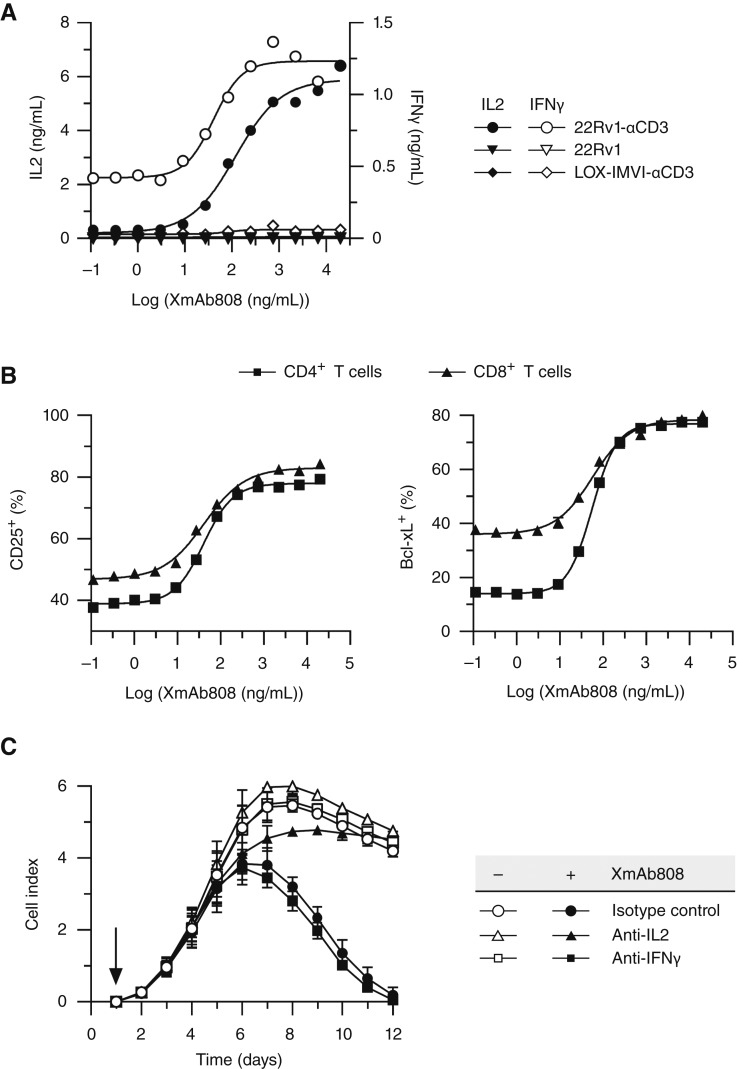
CD28 costimulation drives activation and survival of T cells, leading to IL2-dependent killing of cancer cells. Target cells were cocultured with human T cells (E:T = 1:1) and treated with XmAb808. **A,** After 24 hours, IL2 (closed symbols) and IFNγ (open symbols) were measured in culture supernatants. **B,** The percentages of CD4^+^ or CD8^+^ T cells that were CD25^+^ or Bcl-xL^+^ from cocultures with 22Rv1-αCD3 cells following 24 hours of treatment are plotted. **C,** Real-time killing of 22Rv1-αCD3 cells in cocultures treated with (closed symbols) or without (open symbols) 1 µg/mL of XmAb808 plus an IL2-neutralizing antibody, an IFNγ-neutralizing antibody, or an isotype control antibody was monitored over time using an xCELLigence Real-Time Cell Analysis System. The arrow indicates the addition of T cells and antibodies at 24 hours after cancer cells were plated. Data are plotted as single points in **A** and **B** and as mean ± SEM of three replicates in **C**.

We then explored the T-cell costimulatory properties of XmAb808 with human carcinoma cell lines by engineering B7-H3^+^ 22Rv1 prostate cancer cells and B7-H3–negative LOX-IMVI melanoma cells to express the same anti-CD3 antibody used in the HEK293-αCD3 cells. XmAb808 dose-dependently induced IL2 and IFNγ release from T cells cocultured with 22Rv1-αCD3 but not parental 22Rv1 cells (Fig. 2A), illustrating the Signal 1 requirement for costimulatory activity. In addition, T cells did not secrete IL2 and IFNγ in B7-H3–negative LOX-IMVI-αCD3 cocultures, even in the presence of the same Signal 1. Furthermore, XmAb808 dose-dependently activated both CD4^+^ and CD8^+^ T cells (measured by CD25 expression) cocultured with 22Rv1-αCD3 cells and stimulated their survival (measured by Bcl-xL expression; Fig. 2B), in agreement with known outcomes of CD28-mediated T-cell costimulation (7, 25). These results demonstrate that B7-H3 engagement by XmAb808 provides tumor antigen–targeted Signal 2 that results in markedly enhanced T-cell responses compared with TCR engagement alone.

An anticipated outcome of costimulation of T cells with cancer cells is enhanced T cell–mediated target cell killing. XmAb808 stimulated T cells to kill 22Rv1-αCD3 cells, and neutralization of either IL2 or IFNγ suggested that IL2 contributed to T cell–mediated killing, whereas IFNγ did not (Fig. 2C). Of note, the enhanced T cell–mediated cytotoxic effects of XmAb808 emerged after 6 days of coculture, implying that costimulation triggered prolonged T-cell responses, such as enhanced differentiation and proliferation. In line with this hypothesis, IL2 supports T-cell proliferation, survival, and differentiation of naïve T cells into effector and memory cells, a process that requires several days (26).

### XmAb808 enhances CD8^+^ T-cell memory responses *in vitro*

**Figure 3.**
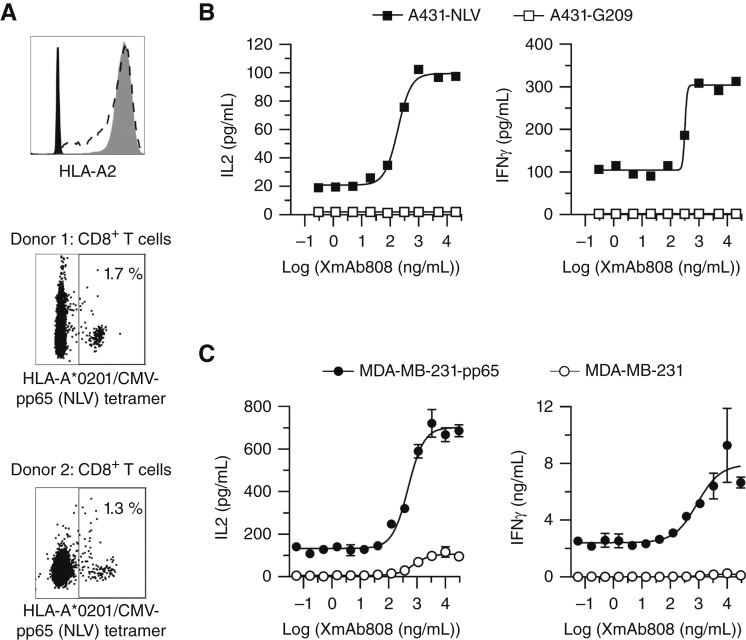
XmAb808 enhances T-cell recall responses to endogenous pMHC ligands. **A** (top graph), Levels of HLA-A2 on A431-β2M-null cells expressing a fusion protein of β2M and HLA-A*0201 fused to CMV pp65 NLV peptide (A431-NLV, dashed black) or melanoma G209 peptide (A431-G209, solid gray) were compared with the signal from unstained cells (solid black). **A** (two bottom graphs), PBMCs from two unique HLA-A*0201 CMV–seropositive donors were stained with HLA-A*0201-CMV-pp65 (NLV) tetramers; the percentage of tetramer+ CD8^+^ T cells is shown. **B,** A431-NLV or A431-G209 cells were cocultured with CD3^+^ T cells from donor 1 at an E:T ratio of 25:1 and treated with XmAb808. IL2 and IFNγ were measured in the supernatants 24 hours after treatment. **C,** Parental MDA-MB-231 cells or MDA-MB-231 cells expressing the CMV pp65 protein (MDA-MB-231-pp65) were cocultured with CD3^+^ T cells from donor 2 at an E:T ratio of 10:1 and treated with XmAb808. IL2 and IFNγ were measured in the supernatants 24 hours after treatment. Data are represented as the mean ± SEM of three replicates.

The anti-CD3 antibody expressed on our engineered cancer cell lines produces homogeneous and high-affinity (∼4 nmol/L K_D_) TCR engagement as a source of Signal 1, in marked contrast to the heterogeneous lower densities and much lower affinities (typically 1–100 μmol/L *K*_*D*_ range) of canonical pMHC–TCR interactions (27). To investigate the costimulatory effects of XmAb808 when such weak native interactions provide Signal 1, we developed an immune recall assay that reproduces a physiologic pMHC-mediated T-cell response to pp65, the immunodominant antigen abundant in CMV infection (19). CMV-reactive T cells are a reasonable model for TILs because both have undergone repeated *in vivo* exposure to antigens and inflammatory signals (28). Based on HLA-A*0201/NLVPMVATV (NLV peptide) tetramer staining, 1.3% to 1.7% of CD8^+^ T cells from two CMV-seropositive donors recognized the pp65 peptide (Fig. 3A) in contrast to the presumed 100% of T cells engaging with the clonal anti-CD3–expressing cell lines used above.

To generate a homogeneous pMHC–TCR Signal 1, we expressed specific pp65 NLV or control gp100 pMHCs on B7-H3^+^ A431 cancer cells that lack endogenous surface MHC expression because of CRISPR-mediated deletion of the *β2M* gene. This approach enables defined peptide presentation as a fusion protein of HLA-A*0201 with either the CMV pp65 NLV peptide (on A431-NLV cells) or the control gp100 (melanoma-associated) IMDQVPFSV peptide (on A431-G209 cells). Because of the *β2M* deletion, no sources of Signal 1–mediated allogeneicity exist in these cell lines. To allow direct comparison between the two cell lines, we selected A431-NLV and A431-G209 clones with similar HLA-A levels (Fig. 3A). We then cocultured these engineered A431 cells with CD3-enriched T cells from a human CMV-seropositive donor in which 1.7% of CD8^+^ T cells recognized the pp65 NLV peptide. This donor had an HLA-A*0201 genotype, which provided the required HLA match between target and effector cells [Fig. 3A (donor 1)].

XmAb808 stimulated dose-dependent IL2 (EC_50_: 186 ng/mL) and IFNγ (EC_50_: 321 ng/mL) release from HLA-matched T cells cocultured with A431-NLV cells but not with A431-G209 cells (Fig. 3B). This requirement for pMHC–TCR engagement further demonstrates that XmAb808 is not superagonistic and requires a cognate Signal 1 for its B7-H3–mediated costimulatory activity. Even in the absence of costimulation by XmAb808, T cells secreted more basal IL2 and IFNγ in A431-NLV cocultures than in A431-G209 cocultures (Fig. 3B), suggesting that Signal 1 was supplied by NLV peptide presentation to CMV-reactive memory T cells. The stimulation of both IL2 and IFNγ by XmAb808, and at similar potencies, is notable because these cytokines enhance T cell–mediated antitumor responses through non-overlapping mechanisms. As noted above, IL2 supports T-cell proliferation, survival, and differentiation (26), whereas IFNγ enhances antitumor immunity by promoting antigen presentation and by directly killing cancer cells (29).

CD80/CD86-mediated CD28 costimulation reduces the threshold of T-cell activation, increasing T-cell responsiveness to low-affinity pMHCs (30). We therefore investigated whether tumor antigen–targeted costimulation by XmAb808 could enhance a physiologically relevant and more diverse Signal 1 provided by endogenous pMHC on target cancer cells. We developed another antigen recall assay in which the entire pp65 protein was processed and presented as heterogeneous pMHCs on HLA-A*0201^+^ B7-H3^+^ MDA-MB-231 breast cancer cells (MDA-MB-231-pp65). We reasoned that MHC presentation of diverse CMV-derived epitopes at physiologic expression levels by these cells would better model endogenous antigen presentation by an intact immune system as compared with the high-level presentation of a single unique CMV-derived peptide in the A431-NLV model.

MDA-MB-231-pp65 cells were cocultured with T cells from a different CMV-seropositive donor having an HLA-A*0201 genotype and with 1.3% of CD8^+^ T cells recognizing the pp65 NLV peptide based upon HLA-A*0201/NLV tetramer staining [Fig. 3A (donor 2)]. Similar to the A431 model (Fig. 3B), in the absence of XmAb808, T cells in MDA-MB-231-pp65 cocultures secreted more basal IL2 and IFNγ than those in parental MDA-MB-231 cocultures (Fig. 3C), suggesting that CMV-derived pp65 antigens were processed and presented to CMV-reactive memory T cells. XmAb808 enhanced this recall response in a dose-dependent manner, stimulating both IL2 (EC_50_: 486 ng/mL) and IFNγ (EC_50_: 883 ng/mL) release in MDA-MB-231-pp65 cocultures, with little to no response in MDA-MB-231 cocultures (Fig. 3C). XmAb808 stimulated a low-level IL2 response (EC_50_: 767 ng/mL) in the absence of CMV peptide presentation, which may have been an alloreactive effect mediated by other mismatched HLA alleles that activated Signal 1 in a small fraction of T cells. The potencies of IL2 and IFNγ release with A431-NLV or MDA-MB-231-pp65 were comparable, suggesting effective costimulation regardless of the model system used.

### XmAb808 enhances the activity of CD3 T-cell engagers

**Figure 4.**
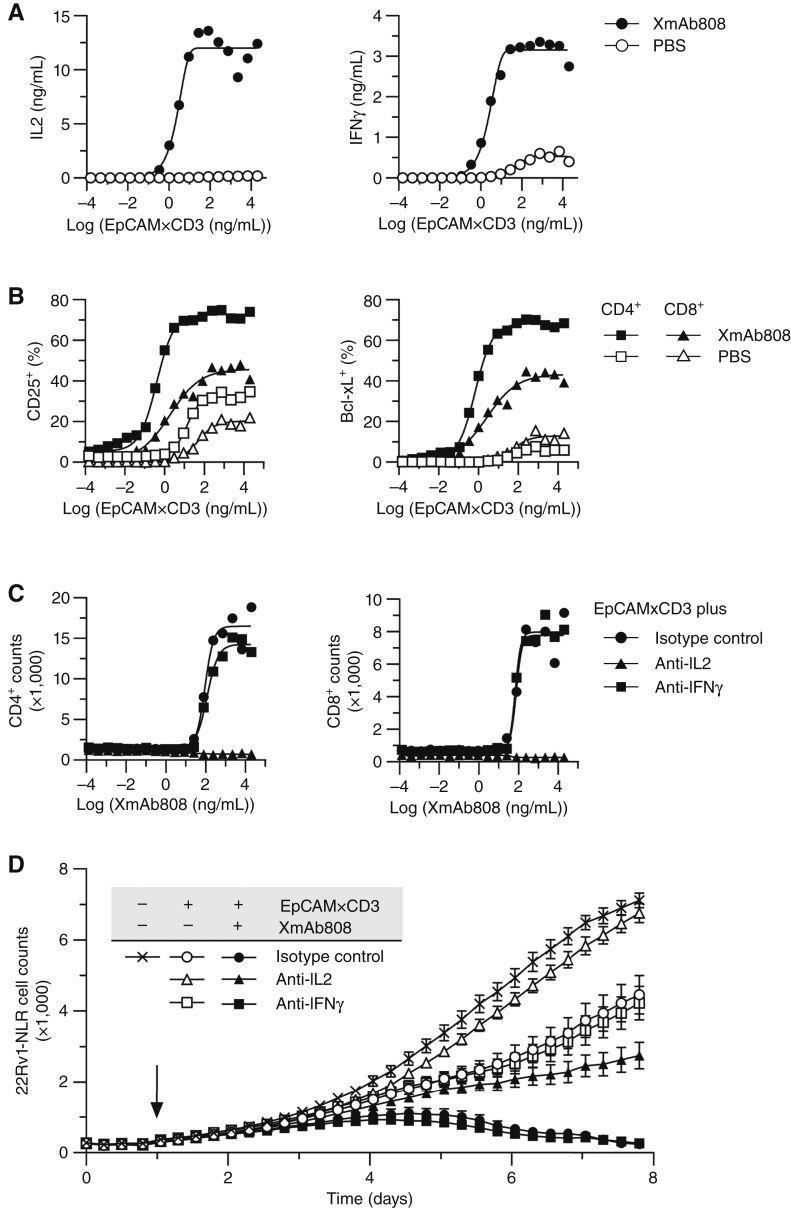
XmAb808 enhances the activity of an EpCAM×CD3 TCE *in vitro*. **A** and **B,** 22Rv1-NLR cells were cocultured with T cells at an E:T ratio of 10:1 and treated with a dose titration of EpCAM×CD3 with (closed symbols) or without (open symbols) 1 μg/mL of XmAb808. **A,** After 24 hours, IL2 and IFNγ secretion was measured, and **B,** CD25^+^ or Bcl-xL^+^ T cells were counted. CD25^+^ or Bcl-xL^+^ cell counts are presented as a percentage of total CD4^+^ or CD8^+^ T cells following 24 hours of treatment. For cytokine measures, data are represented as the means of two replicates. **C,** 22Rv1-NLR cells were cocultured with T cells at an E:T ratio of 1:1 and treated with a dose titration of XmAb808 and 1 ng/mL of EpCAM×CD3; 10 μg/mL of an IL2-neutralizing, an IFNγ-neutralizing, or an isotype control antibody was also added. After 5 days, CD4^+^ and CD8^+^ T cells in 100 μL were counted with flow cytometry. **D,** The viability of 22Rv1-NLR cells in coculture with T cells at an E:T ratio of 1:1 was monitored in real time using an Incucyte Live-Cell Analysis System. Cocultures were treated with 10 μg/mL of EpCAM×CD3, 1 μg/mL of XmAb808, and/or 10 μg/mL of an IL2- neutralizing, an IFNγ-neutralizing, or an isotype control antibody, as indicated. The arrow indicates when T cells and antibodies were added. Data are represented as means ± SEM; *n* = 3.

We reasoned that Signal 2 provided by XmAb808 would effectively combine with CD3 T-cell engagers (TCE) that generate tumor antigen–targeted Signal 1 in T cells. This combination of two tumor-targeted bispecific antibodies (one binding CD28 to replicate costimulation and the other binding CD3 to replicate TCR engagement) should reproduce two critical components of the synapse between cancer cells and T cells and thus enhance the immune response in the TME. This therapeutic approach requires that cancer cells express both antigens targeted by the CD3 and CD28 bispecific antibodies. To test this concept, we assessed the immunostimulatory effects of XmAb808 combined with an EpCAM×CD3 bispecific antibody in cocultures of T cells with B7-H3^+^ EpCAM^+^ 22Rv1 human prostate carcinoma cells. EpCAM×CD3 alone modestly increased IFNγ but not IL2 secretion dose-dependently (Fig. 4A), whereas the addition of 1 μg/mL of XmAb808 to EpCAM×CD3 markedly increased the secretion of IL2 and IFNγ. XmAb808 alone did not induce IL2 or IFNγ, again demonstrating that its costimulatory effects require Signal 1. By 24 hours of coculture, the combination increased activated CD25^+^ T cells along with T cells expressing the survival marker Bcl-xL (Fig. 4B). These XmAb808 properties are consistent with the known physiologic effects of CD80/CD86-mediated CD28 signaling that lead to Bcl-xL upregulation (25). Given these results, we hypothesized that XmAb808 combined with EpCAM×CD3 would stimulate T-cell proliferation, leading to enhanced targeted cytotoxic activity. We therefore tested the effect of a dose titration of XmAb808 combined with a fixed (1 ng/mL) EpCAM×CD3 concentration on T-cell proliferation after 5 days. EpCAM×CD3 alone did not increase T-cell counts (Fig. 4C). However, the addition of XmAb808 dose-dependently promoted an increase in CD4^+^ and CD8^+^ T cells. Neutralization of IL2 signaling but not of IFNγ signaling eliminated this T-cell expansion.

We hypothesized that the pharmacodynamic effects resulting from the combination of XmAb808 with EpCAM×CD3 would manifest in enhanced T cell–mediated killing over the course of several days. Therefore, we monitored T cell–mediated killing of 22Rv1 cells over time. 22Rv1 cells were labeled with Nuclight-red (22Rv1-NLR) to kinetically track T cell–mediated killing in real time. Although 10 µg/mL of EpCAM×CD3 alone promoted some T-cell killing of 22Rv1 cells, the addition of 1 μg/mL of XmAb808 markedly enhanced this effect, reducing target cell counts to baseline (Fig. 4D). As observed in Fig. 2C, the onset of enhanced T cell–mediated killing occurred several days after antibody treatment, suggestive of a sustained IL2 response that stimulated proliferation, differentiation, and survival of T cells. In agreement with the T-cell proliferation results, neutralization of IL2 but not IFNγ suppressed T cell–mediated killing stimulated by EpCAM×CD3 alone and in combination with XmAb808 (Fig. 4D).

Taken together, these data suggest that XmAb808 reproduces CD28 costimulation by amplifying TCR-mediated T-cell responses, leading to differentiation of naïve T cells into effector and memory cells (26). Two mechanisms by which CD28 stimulation improves antitumor activity are through the promotion of T-cell expansion through IL2/CD25 signaling and the enhancement of T-cell survival through upregulation of the antiapoptotic protein Bcl-xL. In this model, XmAb808 again had no activity in the absence of EpCAM×CD3, demonstrating that maximal T-cell responses require the formation of immune synapses incorporating both Signal 1 and Signal 2 elements.

Next, we explored the specific T-cell subsets (naïve, central memory, effector memory, and terminally differentiated effector memory) costimulated by XmAb808. We measured intracellular T-cell IL2 expression in cocultures of B7-H3^+^ A431 cells and CD3-enriched T cells treated with XmAb808 alone or combined with a B7-H3×CD3 TCE for 24 hours. This CD3 TCE was engineered to avoid competition with XmAb808 for B7-H3 binding. B7-H3×CD3 alone stimulated minimal IL2 production in any T-cell subset, whereas the addition of XmAb808 significantly increased IL2-expressing CD4^+^ and CD8^+^ T cells across all four subsets (**Supplementary Fig. S1**). These findings demonstrate that the combination of XmAb808 and B7-H3×CD3, but not B7-H3×CD3 alone, stimulates all major subsets of CD4^+^ and CD8^+^ T cells.

### XmAb808 reactivates exhausted T cells

Given that T cells in the tumor environment are frequently exhausted (31), we assessed if XmAb808 could rejuvenate hyporesponsive T cells, using a well-established model in which exhaustion is triggered by prolonged incubation of T cells with αCD3/αCD28 antibody–coated beads in the presence of IL2 (32, 33). Following this combined Signal 1/Signal 2 chronic overstimulation, the exhaustion markers PD-1, CTLA4, LAG3, TIM3, and TOX were significantly upregulated in both CD4^+^ and CD8^+^ T cells compared with untreated controls (**Supplementary Fig. S2A**). We then cocultured these exhausted T cells with B7-H3^+^ A431 cells and treated them with B7-H3×CD3 (providing Signal 1) alone or in combination with XmAb808 (providing Signal 2). Whereas B7-H3×CD3 elicited minimal IL2 and IFNγ secretion, the addition of XmAb808 significantly and dose-dependently increased the secretion of both cytokines (**Supplementary Fig. S2B**). These findings indicate that adding XmAb808 to a CD3 TCE revives exhausted T cells, suggesting that such combinations may enhance antitumor activity.

### XmAb808 enhances *in vivo* antitumor activity of a CD3 bispecific TCE

**Figure 5.**
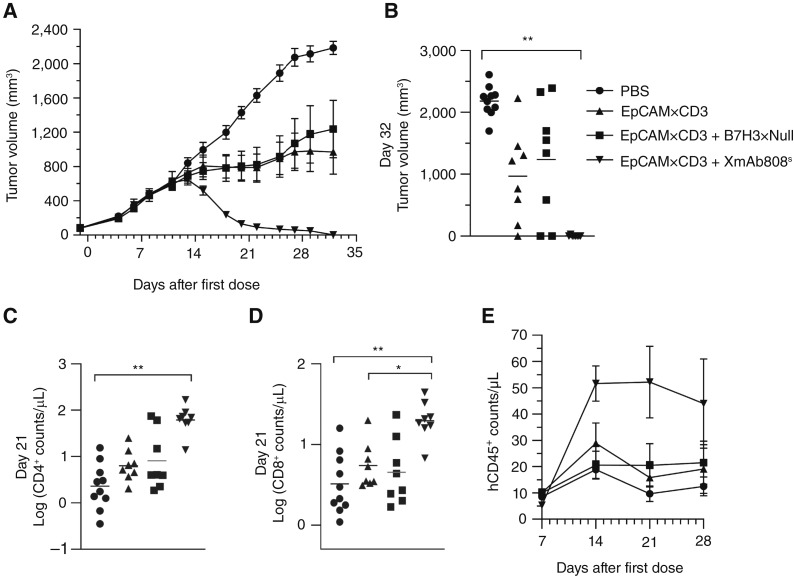
An XmAb808 analogue enhances antitumor activity of an EpCAM×CD3 TCE in human PBMC-engrafted mice. NSG-MHC I/II DKO mice were intradermally inoculated with HPAF-II cells. After 2 weeks, palpable tumors formed, and mice were then engrafted with human PBMCs together with 5 mg/kg of EpCAM×CD3 alone or in combination with 1 mg/kg of B7-H3×Null or 1 mg/kg of XmAb808^s^ weekly. **A,** Tumor volumes are shown over time as mean ± SEM with *n* = 8–10 mice/group. **B,** Tumor volumes of individual mice at the end of the study. Each horizontal line represents mean values. **C** and **D,** Human T-cell counts in peripheral blood after 21 days of treatment. Each horizontal line represents the geometric mean. **E,** hCD45^+^ cells counted in the peripheral blood are shown over time as mean ± SEM with *n* = 8–10 mice/group. For **B–D**, asterisks denote statistical significance:*, *P* < 0.05; **, *P* < 0.01. XmAb808^s^ is a surrogate B7-H3×CD28 bispecific identical to XmAb808 except it lacks the half-life–extending mutations in its Fc domain.

The *in vitro* T-cell costimulatory properties of XmAb808 suggest that it may enhance the antitumor efficacy of other immunotherapies in mouse models. We first explored the combination of targeted CD28 costimulation with a tumor-targeted Signal 1 provided by the EpCAM×CD3 bispecific antibody. Costimulation was provided by XmAb808^s^, a surrogate B7-H3×CD28 bispecific antibody identical to XmAb808 except that it lacks the Fc-domain M428L/N434S mutations, which increase serum half-life in humans but may reduce half-life in mice (34). Immunocompromised NSG-MHC I/II DKO mice were inoculated with B7-H3^+^ EpCAM^+^ HPAF-II human pancreatic cancer xenografts, engrafted with human PBMCs following the formation of palpable tumors, and then dosed weekly with antibodies. Tumor volume reductions relative to PBS controls occurred by ∼15 to 18 days after the start of dosing, with the XmAb808^s^ and EpCAM×CD3 combination causing a sharp reduction in tumor growth that was superior to EpCAM×CD3 monotherapy, as well as complete tumor growth suppression by 32 days postengraftment (Fig. 5A and B). Human T-cell proliferation correlated with antitumor efficacy; at day 21, XmAb808^s^ plus EpCAM×CD3 increased CD4^+^ and CD8^+^ T-cell counts by 9.6-fold and 3.6-fold, respectively, compared with EpCAM×CD3 alone (Fig. 5C and D). To confirm that costimulation was required for the antitumor effect of XmAb808^s^ and to exclude the possibility that its binding to B7-H3 on cancer cells in the absence of T-cell engagement might enhance the efficacy of EpCAM×CD3, we designed a bispecific antibody lacking the anti-CD28–binding domain of XmAb808^s^ (B7-H3×Null). This control antibody, in marked contrast to XmAb808^s^, did not increase the antitumor activity of EpCAM×CD3 (Fig. 5A) or boost T-cell counts (Fig. 5C and D), highlighting the requirement for CD28 engagement to generate tumor-targeted costimulation.

The delayed tumor reduction in the EpCAM×CD3 and XmAb808^s^ combination group (Fig. 5A) can be attributed to the time required for sufficient human T-cell proliferation to exert an antitumor effect. We counted human CD45^+^ cells in peripheral blood weekly throughout the study as a surrogate for human T-cell proliferation. Counts were initially low at 7 days postengraftment and progressively increased over time in mice receiving the combination of EpCAM×CD3 and XmAb808^s^ compared with the EpCAM×CD3 alone group (Fig. 5E). This enhanced human leukocyte proliferation coincides with the antitumor activity of the combination therapy, suggesting that the increased number of functional human T cells contributes to the observed tumor regression at days 15 to 18.

### XmAb808 enhances alloreactivity and combines with an anti–PD-1 to suppress growth of tumor xenografts

**Figure 6.**
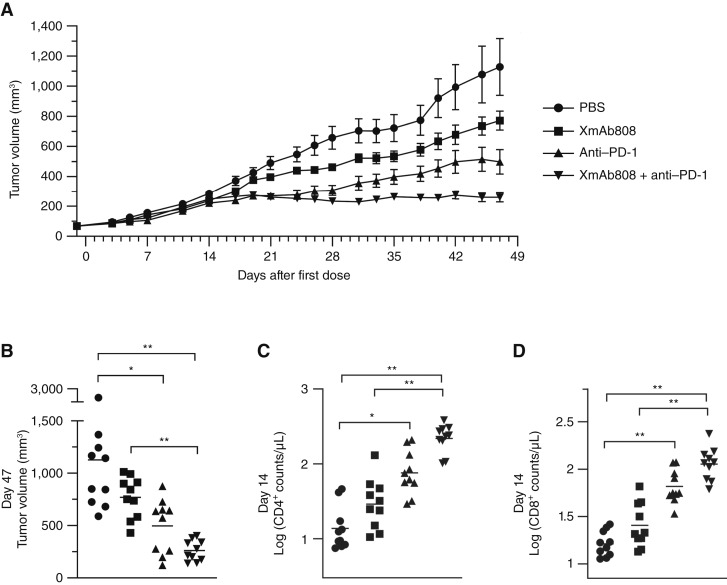
XmAb808 combines with an anti–PD-1 antibody to suppress tumor growth in human PBMC-engrafted mice. NSG-MHC I/II DKO mice were intradermally inoculated with MDA-MB-231-pp65 cells. After 18 days, palpable tumors formed, and mice were engrafted with human PBMCs together with 3 mg/kg of XmAb808 and/or 3 mg/kg of anti–PD-1 weekly. **A,** Tumor volumes are shown over time as mean ± SEM of 10 mice/group. **B,** Tumor volumes of individual mice at the end of the study. Each horizontal line represents mean values. **C** and **D,** Human CD4^+^ and CD8^+^ T-cell counts in the peripheral blood after 14 days of treatment. Each horizontal line represents the geometric mean. For **B–D**, asterisks denote statistical significance: *, *P* < 0.05; **, *P* < 0.01.

Because PD-L1/PD-1 signaling attenuates T-cell activation by inhibiting TCR signaling and excluding CD28 from the immune synapse (10, 12, 13, 35–37), we next assessed the antitumor effects of XmAb808 combined with an anti–PD-1 antibody to promote Signal 1 and/or restore CD28 signaling. NSG-MHC I/II DKO mice inoculated with B7-H3^+^ PD-L1^+^ MDA-MB-231-pp65 breast cancer cells were engrafted with human PBMCs and dosed weekly with antibodies. In this model, the Signal 1 required for XmAb808 activity is driven by HLA mismatches between tumor cells and engrafted T cells. Antibody treatment generally inhibited tumor growth versus PBS starting ∼17 days after the first dose (Fig. 6A). Both XmAb808 monotherapy and anti–PD-1 monotherapy reduced tumor volumes, although effects of XmAb808 alone were relatively modest. Whereas anti–PD-1 markedly suppressed tumor growth in only 4 of 10 mice, strikingly, the combination of XmAb808 and anti–PD-1 suppressed tumor growth in all treated mice (Fig. 6A and B). CD4^+^ and CD8^+^ T-cell counts increased earlier than the observed antitumor activity (that is, 14 days after the first dose), with anti–PD-1 expanding CD4^+^ and CD8^+^ T cells 5.5-fold and 4.0-fold relative to PBS (Fig. 6C and D). Again, the combination of XmAb808 and anti–PD-1 stimulated the greatest CD4^+^ and CD8^+^ T-cell expansion, 2.9-fold and 1.7-fold higher than that of anti–PD-1 alone. Even though T cells significantly expanded with combination XmAb808 and anti–PD-1 treatment, symptoms of GVHD such as weight loss did not differ between treatment groups, suggesting that the expanded T cells primarily targeted the tumor rather than the host. Overall, these results suggest that XmAb808 can provide targeted costimulation to T cells in which a functional CD28 signaling pathway has been restored by an anti–PD-1 checkpoint inhibitor, with the combination promoting significant T-cell expansion and antitumor activity.

## Discussion

The essential role of costimulation (Signal 2) in mounting a robust antitumor response was shown in mice over 30 years ago (3, 4). Despite early interest in harnessing costimulation as a cancer immunotherapy (38), to date, no drugs targeting CD28 have received regulatory approval. The clinical failure of TGN1412 (5) firmly established that biologics engaging CD28 must be nonsuperagonistic and also suggested that their potent immune stimulation should be restricted to the TME.

Notwithstanding this early setback, the role of CD28 signaling in antitumor immunity remains indisputable. Notably, two clinically validated targets of immune checkpoint inhibitors are known to suppress T-cell costimulation. CTLA4—the T-cell target of approved immunotherapies ipilimumab and tremelimumab—is a decoy receptor for CD80 and CD86, having higher affinity for these ligands than CD28 itself; thus, such anti-CTLA4 antibodies free CD80 and CD86 to enhance costimulation through CD28 systemically and boost effector T-cell antitumor responses (39). Likewise, PD-1—the target of approved biologics nivolumab, pembrolizumab, and others—suppresses CD28 signaling to an even greater extent than TCR signaling (12, 13). Although neither of these checkpoint inhibitors engage CD28 directly, their primary therapeutic mechanism is enhancement of costimulation, strongly suggesting that biologics that stimulate CD28 signaling in a tumor-targeted manner have potential as a novel class of immunotherapy. This recognition, coupled with new protein engineering tools to generate bispecific antibodies, has renewed interest in therapeutics that focus T-cell costimulation against tumor cells in the TME.

We show here that XmAb808 is one such molecule that recapitulates known outcomes of physiologic CD28-mediated costimulation. It promotes activation, proliferation, and survival of T cells, induces cytokine release from T cells, and ultimately, stimulates greater T cell–mediated killing of B7-H3^+^ target cells in the presence of Signal 1. We also demonstrate that IL2 contributes to these responses. In the 1990s, high-dose IL2 was approved to treat metastatic renal cell carcinoma and metastatic melanoma but has had limited clinical impact due to significant toxicities (40). Since then, efforts have been made to modulate IL2 exposure and its resulting signaling using various therapeutic strategies (40–42). In light of this, our studies indicate that XmAb808 causes localized IL2 secretion, along with upregulation of its receptor (CD25), creating an autocrine positive feedback loop for tumor-reactive T cells. This strategy potentially retains the antitumor properties of IL2 while avoiding the deleterious effects of high-dose systemic administration.

Targeted costimulation need not only rely on neoantigen-derived Signal 1 to enhance antitumor immunity. Signal 1 can be generated artificially via a biologic, such as a tumor-targeted CD3 TCE. Such bispecific antibodies are under intensive clinical investigation and are beginning to show considerable promise for the treatment of solid tumors (43). Notable examples include tebentafusp, a gp100×CD3 TCE targeting HLA-A*0201/gp100 complexes that was recently approved for uveal melanoma (44), and tarlatamab, a DLL3×CD3 TCE that was recently approved for small cell lung cancer (45). Moreover, xaluritamig, a STEAP1×CD3 bispecific antibody, has demonstrated promising activity in metastatic castration–resistant prostate cancer in an early clinical study (46).

Solid tumors may have low levels of TILs (47); therefore, CD28 bispecifics like XmAb808 may be critical combination partners to maximize the efficacy and durability of classic CD3 TCEs. The data discussed herein support this concept, as XmAb808 enhanced antitumor effects of an EpCAM×CD3 bispecific *in vitro* and in mice. The results from our *in vitro* and *in vivo* studies suggest that a combination of XmAb808 (providing a tumor-targeted Signal 2) and a CD3 TCE (providing a Signal 1 targeted to a different antigen on the same tumor) may enhance efficacy or safety more than simply increasing the dose of the CD3 TCE alone. In multiple models presented here, the targeted Signal 2 greatly amplified the potential therapeutic effects of the targeted Signal 1; therefore, although ultimately this hypothesis must be tested in clinical trials, we speculate that combinations of CD28 TCEs such as XmAb808 with CD3 TCEs such as the bispecific antibodies noted above will improve the overall therapeutic index.

We selected B7-H3 as our first CD28 TCE target because it is expressed at high levels across many tumor types yet still detected at lower levels in some normal tissues (11). Given the relative insensitivity of T cells to Signal 2 alone, we anticipate that the well-known OTOT toxicity of CD3 TCEs will not be amplified by XmAb808. Thus, we predict that XmAb808 may combine with diverse CD3 TCEs to amplify T-cell responses that are highly restricted to tumor cells that are double-positive for B7-H3 and each CD3 TCE target. Ideally, tumor antigens selected for CD3 and CD28 TCE combinations should have non-overlapping expression patterns in healthy tissues, increasing tumor specificity and minimizing OTOT toxicity of the combination immunotherapy to maximize the therapeutic index. As examples, XmAb808 may effectively combine with PSMA-, STEAP1-, or KLK2-targeted CD3 TCEs in prostate cancer; an ENPP3-targeted CD3 TCE in renal carcinoma; a CLDN6-targeted CD3 TCE in ovarian carcinoma; or a DLL3-targeted CD3 TCE in small cell lung cancer, as these tumor types express B7-H3 alongside these more specific tumor-associated antigens (11).

Given that checkpoint inhibitors function in part by restoring CD28 signaling, it is unsurprising that XmAb808 combined with an anti–PD-1 was more efficacious in a mouse xenograft tumor model than either agent alone. These results support an ongoing clinical trial (NCT05585034) exploring XmAb808 combined with pembrolizumab for advanced solid tumors (48).

It is also noteworthy that the CD28 bispecific antibody format is modular. Analogous to using similar or identical CD3-binding domains in multiple CD3 TCEs, the same monovalent CD28-binding domain in XmAb808 can be readily coupled with binding domains for other tumor-specific antigens to generate new clinical candidates against a range of cancers (49). We incorporated a bivalent B7-H3–binding domain in XmAb808 to drive high-avidity binding to tumor cells, coupled with a monovalent, lower-affinity CD28-binding domain to minimize engagement of T cells outside of the TME. We believe that this bivalent/monovalent “2+1” common light-chain format can likewise generate avid targeting of other tumor antigens. We also speculate that this CD28 TCE format could be used to target tumor antigens too broadly expressed for safe use of CD3 TCEs, a class of immunotherapies known for the induction of cytokine release syndrome. Such broadly expressed tumor antigens may be more amenable to targeting with a CD28 TCE, with the natural avidity of the 2+1 format increasing the pool of antigens with therapeutic potential beyond the limited number of tumor-associated targets interrogated using CD3 TCEs.

In conclusion, our results suggest that targeted T-cell costimulation using bispecific antibodies that co-engage CD28 with a tumor-associated antigen has potential as a novel cancer immunotherapy. Clinical investigations of XmAb808 as a monotherapy and in combination with pembrolizumab are ongoing (NCT05585034) to determine if targeted costimulation has clinical potential as a treatment for B7-H3^+^ solid tumors (48).

## Supplementary Material

Supplementary Figure S1 Supplementary Figure S1: XmAb808 Combines With TCEs to Promote IL2 secretion in Naive, Central Memory, Effector Memory, and TEMRA T Cells.

Supplementary Figure S2 Supplementary Figure 2. XmAb808 Combines With a B7-H3×CD3 TCE to Stimulate IL2 and IFNγ Release From Chronically Stimulated, Exhausted T Cells.
