# Supplementary material for: A B7-H3–Targeted CD28 Bispecific Antibody Enhances the Activity of Anti–PD-1 and CD3 T-cell Engager Immunotherapies
Source: Mol Cancer Ther. 2024 Sep 20;24(3):331–44. doi: 10.1158/1535-7163.MCT-24-0327 (PMC11876962; doi:10.1158/1535-7163.MCT-24-0327)
Supplement: Supplementary Figure S1 — XmAb808 Combines With TCEs to Promote IL2 secretion in Naive, Central Memory, Effector Memory, and TEMRA T Cells. [file mct-24-0327_supplementary_figure_s1_supps1.pdf]

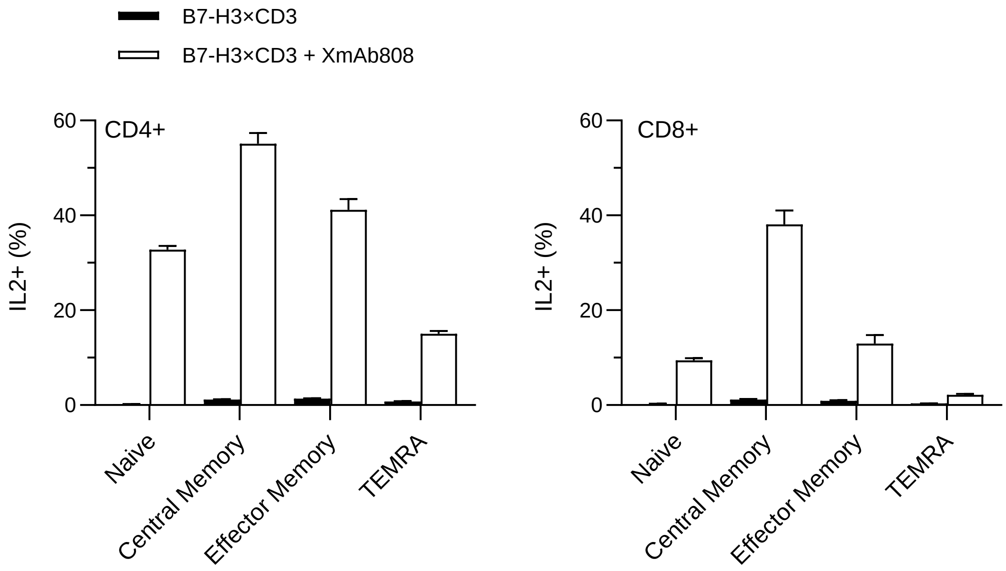

Supplementary Figure S1: XmAb808 Combines With TCEs to Promote IL2 secretion in Naive, Central Memory, Effector Memory, and TEMRA T Cells.

A431 cells were cocultured with T cells at an E:T ratio of 10:1 and treated with 1 μg/mL of B7-H3×CD3 with or without 1 μg/mL of XmAb808. After 24 hours, cells were stained for intracellular IL2. IL2+ cells were counted within CD4+ and CD8+ subsets, including naive (CD45RA+ CCR7+), central memory (CD45RA- CCR7+), effector memory (CD45RA- CCR7-), and TEMRA (CD45RA+ CCR7-) subsets. Percentages shown are IL2+ cells detected in each CD4+ T-cell subset on the left graph and in each CD8+ T-cell subset on the right graph. Data are means ± SEM; n = 3.
