# Supplementary material for: A B7-H3–Targeted CD28 Bispecific Antibody Enhances the Activity of Anti–PD-1 and CD3 T-cell Engager Immunotherapies
Source: Mol Cancer Ther. 2024 Sep 20;24(3):331–44. doi: 10.1158/1535-7163.MCT-24-0327 (PMC11876962; doi:10.1158/1535-7163.MCT-24-0327)
Supplement: Supplementary Figure S2 — Supplementary Figure 2. XmAb808 Combines With a B7-H3×CD3 TCE to Stimulate IL2 and IFNγ Release From Chronically Stimulated, Exhausted T Cells. [file mct-24-0327_supplementary_figure_s2_supps2.pdf]

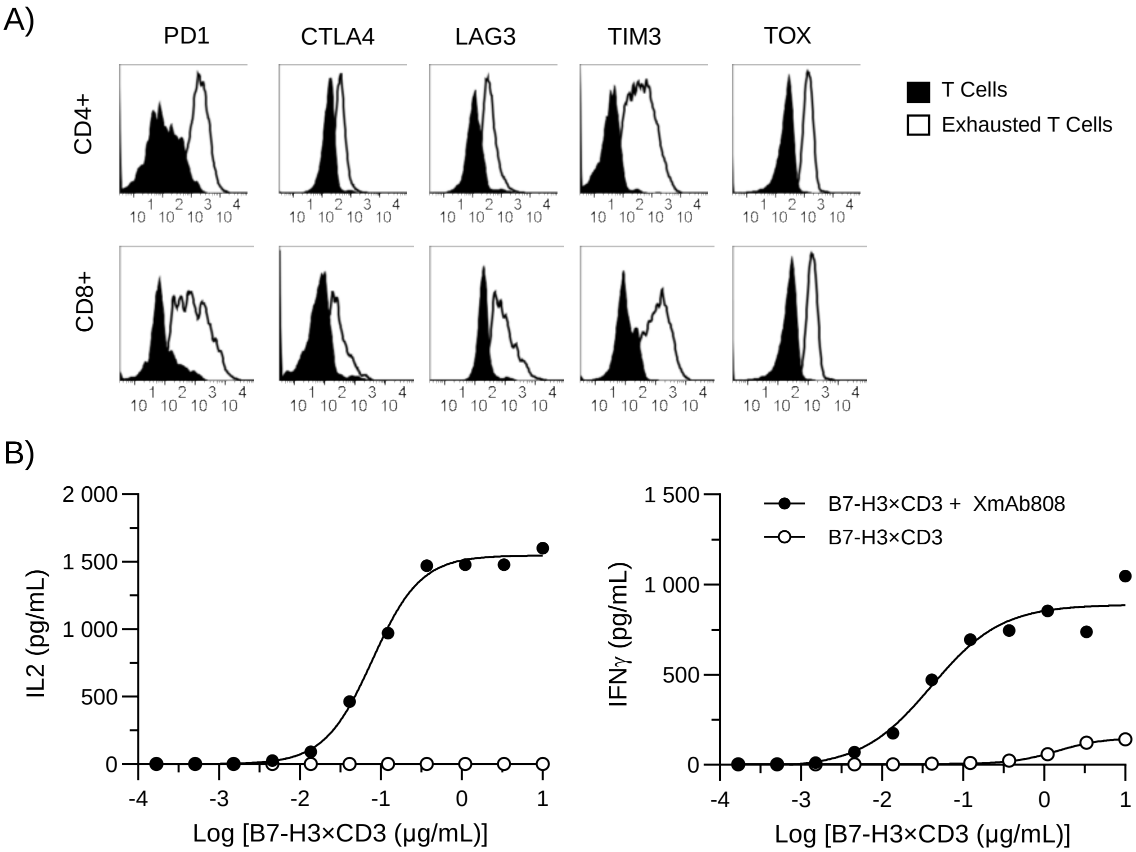

Supplementary Figure 2. XmAb808 Combines With a B7-H3×CD3 TCE to Stimulate IL2 and IFNγ Release From Chronically Stimulated, Exhausted T Cells.

(**A**) CD3+ enriched T cells were stimulated with αCD3/αCD28 beads for one week and then stained for surface checkpoint receptors and intracellular TOX expression. Histograms show resting (solid) and stimulated (open) CD4+ (top row) and CD8+ (bottom row) T cells. (**B**) Stimulated T cells shown in **(A)** were cocultured with A431 cells and treated with 1 μg/mL of XmAb808 and a dose titration of B7-H3×CD3, then IL2 and IFNγ were measured 24 hours after treatment. Each point represents data from a technical singlet.
